# Spontaneous cervical artery dissection is associated with a distinct peripheral immune cell signature

**DOI:** 10.1371/journal.pone.0340592

**Published:** 2026-01-22

**Authors:** Carolin Beuker, Andreas Schulte-Mecklenbeck, Timo Wirth, Ilka Kleffner, Christian Thomas, Daniel Strunk, Antje Schmidt-Pogoda, Catharina C. Gross, Luisa Klotz, Jens Minnerup

**Affiliations:** 1 Department of Neurology, University of Münster, Münster, Germany; 2 Department of Neurology, University Hospital Knappschaftskrankenhaus, Ruhr University Bochum, Bochum, Germany; 3 Institute of Neuropathology, University of Münster, Münster, Germany; 4 Department of Neurology, Philipps-University Marburg, Germany; 5 Department of Neurology, University Hospital Schleswig-Holstein, Campus Lübeck, and University of Lübeck, Lübeck, Germany; Rutgers: Rutgers The State University of New Jersey, UNITED STATES OF AMERICA

## Abstract

**Objectives:**

Despite being a major cause of ischemic stroke in young adults, the biological underpinnings of cervical artery dissection (CeAD) remain poorly defined. Recent data implicate immune activation as a potential contributor. We aimed to determine whether patients with CeAD display a distinct peripheral immune signature, which may provide insights into pathogenic inflammatory processes.

**Methods:**

Peripheral blood mononuclear cells (PBMCs) from patients with spontaneous CeAD (n = 7 without and n = 11 with ischemic stroke) and ten age-matched healthy controls were analyzed via multi-color flow cytometry. Immune cell composition and activation markers were assessed, and sparse partial least squares discriminant analysis (sPLS-DA) was employed to identify CeAD-associated immune features. A secondary comparison with ischemic stroke controls was included to assess the specificity of identified immune alterations.

**Results:**

Compared to healthy controls, CeAD patients displayed increased frequencies of CD4 ⁺ T cells and decreased natural killer T (NKT) cells. sPLS-DA demonstrated clear separation of CeAD and control immune profiles, driven by increased CD28 expression on naïve CD8 ⁺ T cells, NKp46 on NK cells, and IL-2Rα (CD25) on myeloid dendritic cells (mDC2). Elevated granzyme K in naïve CD8 ⁺ T cells indicated enhanced cytotoxic potential, while regulatory T cells were diminished. These alterations were largely preserved when compared to ischemic stroke controls, suggesting CeAD-specific immune activation. No microbial pathogens were detected by untargeted metagenomic sequencing.

**Discussion:**

CeAD is associated with a distinct peripheral immune signature characterized by enhanced cytotoxic activity and reduced regulatory features. These alterations may reflect a post-infectious autoimmune mechanism triggering CeAD or a secondary immune-inflammatory response to vascular injury. Larger, longitudinal studies are needed to clarify causality and assess whether immune modulation could serve as a therapeutic target in CeAD.

## Introduction

Cervical artery dissection (CeAD), affecting the extracranial carotid and vertebral arteries, is a major vascular condition characterized by a separation of the arterial wall layers, leading to intramural hematoma formation and potential luminal narrowing or occlusion [1]. It accounts for up to 20% of ischemic strokes in young and middle-aged adults, making it a leading cause of stroke in this population [2,3]. Early diagnosis and prompt initiation of antithrombotic or anticoagulation therapy are essential to reduce the risk of recurrent ischemic events and long-term neurological sequelae [4].

The etiology of CeAD is multifactorial and not yet fully understood. Both intrinsic factors, such as underlying connective tissue or vascular disorders, and extrinsic triggers, such as cervical trauma, hypertension, migraine have been implicated [1]. Increasing evidence suggests that CeAD may also involve an infectious or inflammatory component. Temporal associations between recent respiratory infections and CeAD onset have been reported, pointing to inflammation as a potential triggering factor [5,6]. Moreover, viral pathogens such as influenza-like illnesses (ILI), varicella-zoster virus (VZV), and COVID-19 have been identified in CeAD patients at higher-than-expected frequencies [7–9]. Proposed pathophysiological mechanisms include endothelial dysfunction, prothrombotic states, and cytokine-mediated vascular inflammation [5,6,10]. Patients with acute CeAD have been found to exhibit significantly elevated white blood cell counts compared to healthy controls or patients with non-CeAD ischemic stroke, potentially reflecting a pre-existing inflammatory state [11]. Notably, the observed decline in CeAD incidence during the COVID-19 pandemic, likely due to reduced respiratory infections following public health interventions, further supports a possible pathogenic role of infection and inflammtion in CeAD [12].

Although preceding infections appear to play a role in the context of CeAD, the underlying mechanisms remain unclear. To further explore the role of the immune system in CeAD, we characterized peripheral immune profiles of affected patients compared to healthy controls.

## Materials and methods

### Standard protocol approvals, registrations, and patient consents

The study was conducted in accordance with the Declaration of Helsinki and approved by the local ethics committee of Münster University (approval number: AZ 2017–148-f-S). Written informed consent was obtained from all participants for peripheral blood sampling and the use of clinical data.

### Study design and participants of the single-center cohort study

This was a prospective, single-center cohort study conducted at the Department of Neurology, Münster University Hospital, between July 2017 and July 2019. The study included patients diagnosed with spontaneous cervical artery dissection (CeAD) who did not experience an ischemic stroke during inpatient evaluation. Patients with CeAD and concurrent ischemic stroke were excluded to avoid stroke-induced alterations in immune cell profiles, which could confound the interpretation of immune responses associated specifically with dissection. Diagnosis was confirmed by the presence of an intramural hematoma, as detected by two independent imaging modalities: (1) eccentric vessel wall enhancement on fat-saturated T1-weighted magnetic resonance imaging (MRI), and (2) eccentric wall thickening on duplex sonography. All patients underwent both imaging modalities, and all scans were independently reviewed by two experienced neuroradiologists blinded to clinical and laboratory data to ensure diagnostic reproducibility. Peripheral blood was collected from CeAD patients without ischemic stroke (n = 7) between day 6 and day 14 after symptom onset, corresponding to the subacute phase of disease. For comparison, a group of patients with ischemic stroke secondary to CeAD (IS; n = 11) was included to evaluate whether immune alterations identified in non-stroke CeAD also occur in the presence of cerebral ischemia. Age-matched healthy donors without a history of cardiovascular or autoimmune disease served as the control group (n = 10). All study participants, including CeAD patients with and without stroke as well as healthy controls, were systematically screened for potential confounders including recent infections, vaccinations, autoimmune and cardiovascular comorbidities, chronic inflammatory conditions, smoking status, and medication use. None of the included individuals reported acute infection, recent vaccination, or immunomodulatory medication use at the time of sampling. Presenting symptoms were recorded during standardized neurological examination and corroborated by patient report at admission.

### Biobanking

Peripheral blood mononuclear cells (PBMC) were isolated from EDTA-blood by density gradient centrifugation using Lymphoprep (Stemcell technologies) and cryoconserved in CTL-medium (Immunospot) in the vapor phase of a liquid nitrogen tank until flow cytometric analysis as previously described [13].

### Flow cytometry

Flow cytometric analysis was performed according to standardized protocols outlined in detail by Gross et al., 2024 [13]. Briefly, PBMC were thawed and distributed across 19 distinct panels of up to 13 fluorochrome-conjugated antibodies, with 2 – 5x10^5^ cells allocated to each individual panel. Partially, intracellular epitopes were detected by treating PBMC with Perm/Fix buffer (BD Biosciences). Functional properties of T cells and NK cells including production of cytokines and degranulation of cytotoxic vesicle were investigated following restimulation. Samples were acquired on a Cytoflex flow cytometer (Beckman Coulter). To ensure proper compensation, we used single-stained, titrated antibody controls for each fluorochrome, and verified that the compensation settings were optimal for our experimental conditions. For markers with non-bimodal distributions, we employed isotype control antibodies with matching fluorophores to determine the optimal gating thresholds as described previously.^13^ Flow cytometry raw data was analyzed by conventional gating using Kaluza (2.3, Beckman Coulter) by at least two independent experts blinded to group allocation of the samples. Proportions of PBMC subsets and marker expressions were quantified and exported, resulting in 575 parameters reflecting the functional immune-status in great detail.

### Metagenomic sequencing

After nucleic acid extraction using the QIAamp Min Elute Virus Spin Kit, first strand and second strand cDNA synthesis was be performed using the ProtoScript II First Strand cDNA Synthesis Kit with Random Primer 6 and the NEBNext Ultra II Non-Directional RNA Second Strand Synthesis Module (New England Biolabs). Resulting cDNA was purified with DNA purification beads (Twist Bioscience) and inspected using the gDNA ScreenTape assay (Agilent). NGS Library preparation was be performed with the Twist Library Preparation EF Kit 2.0 and the Twist Universal Adapter System (Twist Bioscience) using an adjusted fragmentation step and 12 cycles of pre-capture PCR amplification. The resulting multiplex library was loaded onto a MidOutput Flow Cell and sequenced in paired-end mode on the Illumina NextSeq 500/550 platform. In addition to the four CeAD patient samples, two CNS tissue control samples with reactive changes were sequenced in the same run, yielding a comparable sequencing depth (~23 million reads per sample). For downstream analysis using the IDseq pipeline [14], results were filtered according to the following criteria: ≥ 3 NT reads, NT alignment length ≥70 bp, NT Z-score ≥1 (relative to an internal database of 206 cases without infectious etiology), and restricted to bacterial, fungal and viral taxa.

### Statistical analysis

Statistical analysis was performed using R (4.3.2) via R studio (2024.12.1 Build 563) and GraphPad Prism (10.4.1). Sparse partial least squares discriminant analysis (sPLS-DA) [15] was used from the mixOmics package (6.26.0) with ncomp = 2 and keepX = c(10,10). Receiver operating characteristic (ROC) analysis was performed using the pROC package (1.18.5) [16]. P-values were calculated by Kruskal-Wallis with Dunn’s post test.

## Results

### Clinical characteristics of the study population

Baseline demographic and clinical characteristics of the study population are summarized in Table 1. A total of 18 patients with CeAD and 10 age-matched healthy donors (HD) were included. Among the CeAD cohort, 11 patients presented with ischemic stroke and 7 without. The mean age was 48.9 years (SD 14.4) in the CeAD-stroke group, 44.5 years (SD 10.8) in the non-stroke group, and 38.4 years (SD 6.4) among HD. Women accounted for 33.3%, 57.1%, and 40.0% of participants, respectively. The study population exhibited an almost complete absence of cardiovascular risk factors. All dissections occured spontaneously without direct or indirect trauma. The time from symptom onset to clinical presentation ranged from the day of onset up to seven days. Headache, neck pain, and visual disturbances were the most frequent presenting symptoms in non-stroke CeAD, whereas focal neurological deficits (speech disturbances or motor deficits) were observed exclusively in patients with ischemic stroke. Horner’s syndrome occurred more often in the non-stroke group. Most dissections involved the internal carotid artery, with vertebral artery involvement in approximately half of all cases. The degree of vessel stenosis or occlusion varied between groups, with a higher frequency of ≥70% stenosis and total occlusion among stroke patients. Regarding treatment, the majority of patients without stroke received anticoagulation, whereas antiplatelet therapy, either single or dual, was more common among those with ischemic stroke. No deaths occurred during follow-up (median duration 10 months, IQR 6.5–23.5 for non-stroke CeAD; 5 months, IQR 0–17 for stroke CeAD). All patients achieved favorable functional outcomes, with a modified Rankin Scale (mRS) score ≤ 2 at last follow-up. Only one recurrent dissection was recorded.

**Table 1:** Baseline characteristics of study population.

	Patients with CeAD without ischemic stroke (n = 7)	Patients with CeAD with ischemic stroke (n = 11)	HD (n = 10)	P Value*
Demographics				
Age, mean (SD), y	44.5 (10.8)	48.9 (14.4)	38.4 (6.4)	0.45
Women, n (%)	4 (57.1)	4 (33.3)	4 (40.0)	0.71
Comorbidities, n (%)				
Hypertension	0 (0)	3 (25.0)	0 (0)	0.25
Diabetes mellitus	0 (0)	1 (8.3)	0 (0)	0.99
Smoking, current or history	1 (14.3)	2 (16.7)	0 (0)	0.99
Hyperlipidemia	0 (0)	2 (16.7)	0 (0)	0.50
Connective tissue disorder or fibromuscular dysplasia	0 (0)	0 (0)	0 (0)	0.99
Etiology				
spontaneous	7 (100)	11 (100		0.99
secondary to indirect trauma	0 (0)	0 (0)		0
secondary to direct trauma	0 (0)	0 (0)		0
time from symptom onset to presentation days	0, 0-7	0, 0-1.5		0.61
Presenting symptoms/signs				
asymptomatic	0 (0)	0 (0)		0
headache	4 (57.1)	2 (16.7)		0.14
neck pain	3 (42.9)	1 (8.3)		0.63
visual disturbances	4 (57.1)	3 (25.0)		0.33
Horner’s syndrome	3 (42.9)	0 (0)		0.04
pulsatile tinnitus	1 (14.3)	0 (0)		0.39
speech disturbances	0 (0)	4 (33.3)		0.12
motor deficit	0 (0)	5 (41.7)		0.10
dizziness	0 (0)	3 (25.0)		0.25
Diagnostic modality, n (%)				
CTA	1 (14.3)	9 (75.0)		0.01
MRA	6 (85.7)	6 (50.0)		0.32
Degree of occlusion/stenosis, n (%)				
> 70% stenosis	0 (0)	4 (33.3)		0.12
50%−69% stenosis	5 (71.4)	1 (8.3)		0.01
< 50% stenosis	0 (0)	0 (0)		0
total occlusion	1 (14.3)	3 (25.0)		0.99
subtotal occlusion	1 (14.3)	1 (8.3)		0.99
Affected arteries, n (%)				
common carotid	0 (0)	0 (0)		0
internal carotid	7 (100)	6 (50.0)		0.10
internal carotid, bilateral	3 (42.9)	2 (16.7)		0.33
vertebral	3 (42.9)	6 (50.0)		0.99
vertebral, bilateral	2 (28.6)	2 (16.7)		0.99
Carotid and vertebral	3 (42.9)	1 (8.3)		0.25
Treatment, n (%)				
single antiplatelet therapy	1 (14.3)	4 (33.3)		0.60
dual antiplatelet therapy	0 (0)	3 (25.0)		0.25
anticoagulation	6 (85.7)	4 (33.3)		0.07
Recurrent Dissection	1 (14.3)	0 (0)		0.39
Follow-up				
Time of follow-up, median (IQR), months	10, 6.5-23.5	5, 0-17		0.23
Death, n (%)	0 (0)	0 (0)		0
Modified Rankin scale, mRS				
At last follow-up, median (IQR)				
0–2, n (%)	7 (100)	11 (100)		0.99
3–4, n (%)	0 (0)	0 (0)		0
5–6, n (%)	0 (0)	0 (0)		0

SD, standard deviation; *P value for comparisons of patients in all groups.

### Peripheral immune cell composition in CeAD

**Fig 1:**
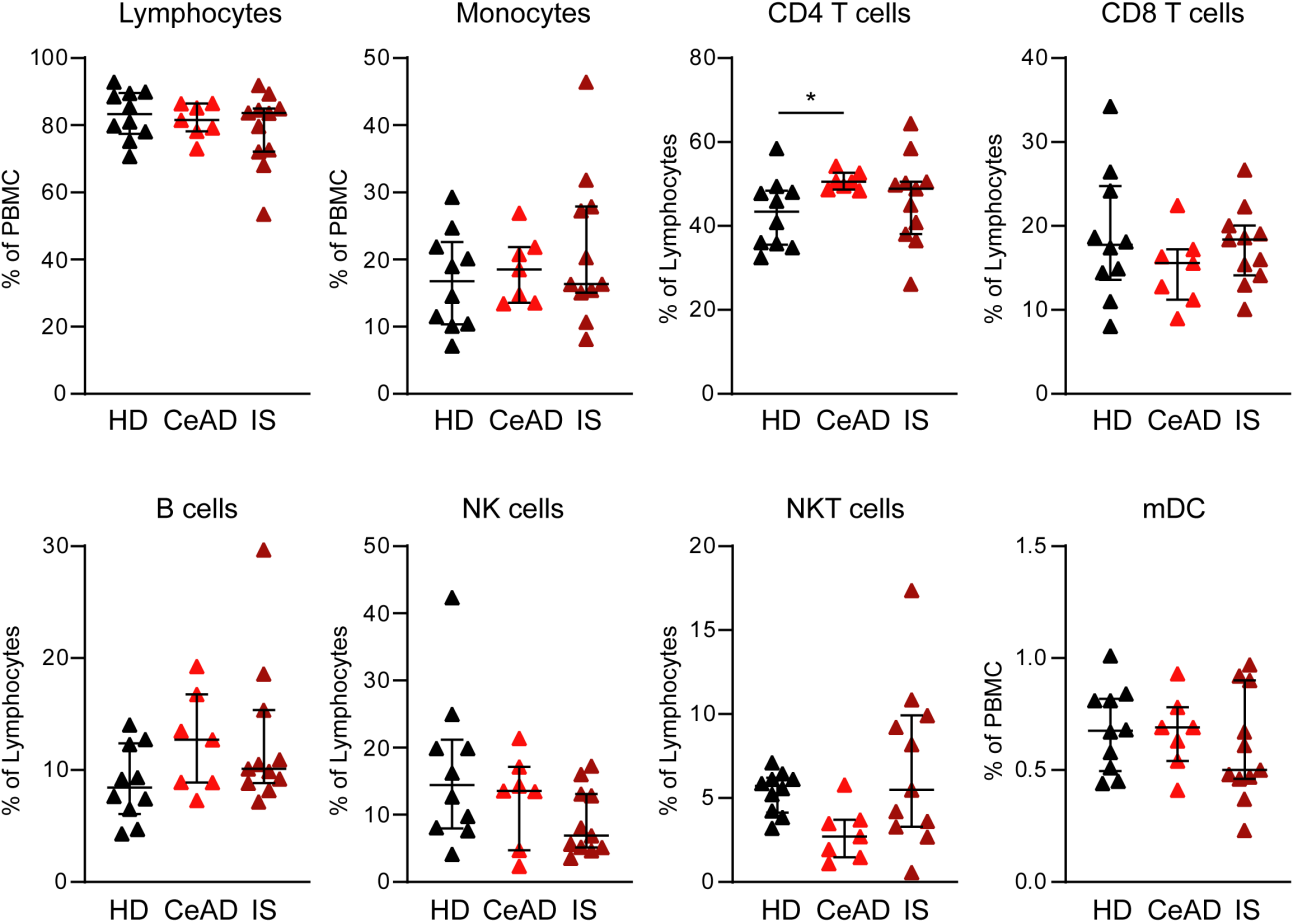
Peripheral immune cell composition in CeAD with and without ischemic stroke. Percentages of major immune cell subsets in PBMCs from healthy donors (HD, black triangles), patients with spontaneous cervical artery dissection without ischemic stroke (CeAD, red triangles), and patients with ischemic stroke secondary to CeAD (IS, dark red triangles) were quantified by flow cytometry. Shown are frequencies of total lymphocytes and monocytes (as % of PBMC), and within the lymphocyte gate: CD4 ⁺ T cells, CD8 ⁺ T cells, B cells, NK cells, and NKT cells (as % of lymphocytes), as well as myeloid dendritic cells (mDC, as % of PBMC). Statistical analysis was performed using Kruskal–Wallis test with Dunn’s post-test for multiple comparisons. Horizontal bars represent mean ± SD (*p < 0.05).

Flow cytometric analysis of peripheral blood mononuclear cells (PBMCs) revealed distinct alterations in major immune cell subsets. Overall lymphocyte and monocyte frequencies, expressed as a percentage of PBMCs, did not differ significantly between HD, patients with spontaneous CeAD without ischemic stroke (CeAD), and patients with ischemic stroke secondary to CeAD (IS) (Fig 1A,1B). Among lymphocyte subpopulations, CeAD patients displayed a higher proportion of CD4 ⁺ T cells compared to HD (p < 0.05, Fig 1C). No significant differences were observed for CD8 ⁺ T cells, B cells, or NK cells between groups (Fig 1D–1F). A reduction of NKT cells was noted in CeAD patients compared to HD, but this did not reach statistical significance after correction for multiple comparisons (Fig 1G). The proportion of myeloid dendritic cells (mDCs) remained comparable across all three groups (Fig 1H). To assess potential sex-related effects, immune cell data were analyzed separately for male and female participants. While several trends, including higher CD4 ⁺ T-cell and lower NKT-cell frequencies in CeAD patients compared to controls, were observable in both sexes, the sex-stratified subgroups were small (especially the male CeAD subgroup) and showed considerable variability. Therefore, these exploratory analyses do not allow firm conclusions regarding sex-independence, and sex-related effects cannot be excluded (Supplementary S1–S2 Figs). Taken together with the overall group-level results, the data indicate a consistent immune profile between CeAD patients with and without stroke, characterized by a tendency toward elevated CD4 ⁺ T cells and lower NKT-cell frequencies relative to healthy controls.

### Multivariate immune profiling reveals CeAD-associated signature

**Fig 2:**
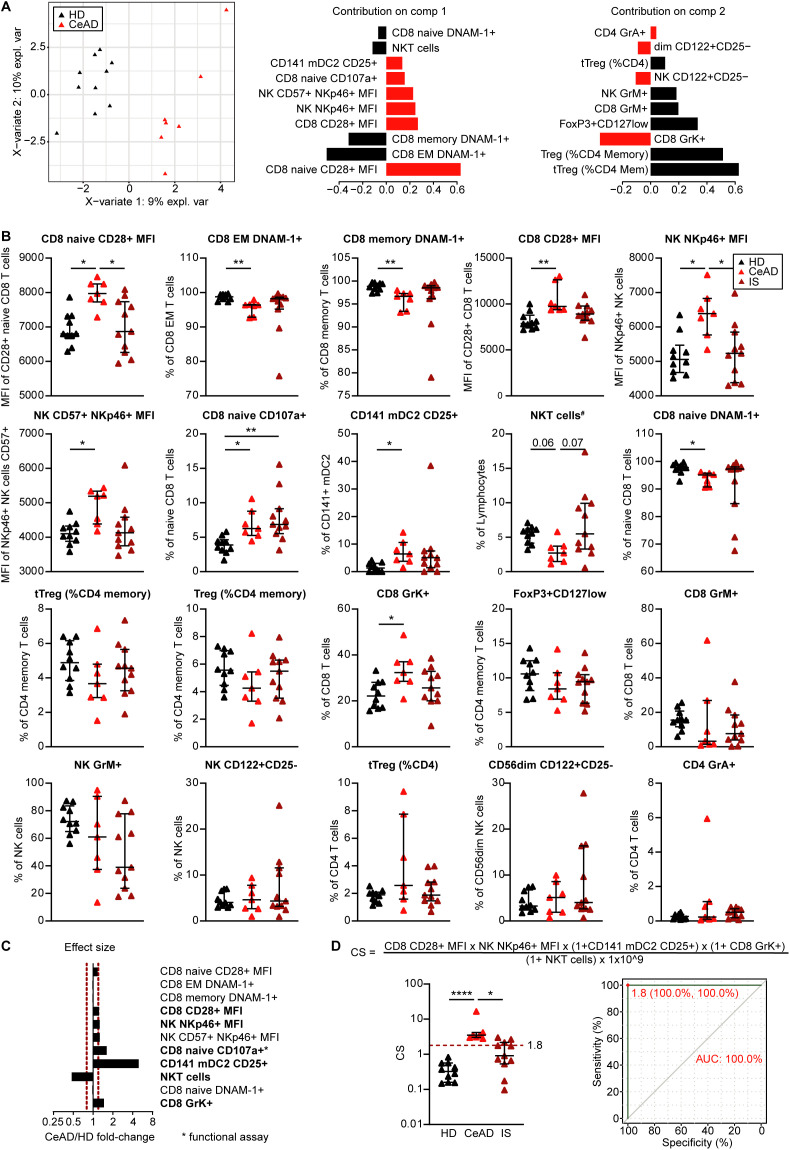
Multidimensional immune profiling reveals a distinct immunophenotype in CeAD. **(A)** Partial least squares discriminant analysis (PLS-DA) of immune cell parameters in CeAD patients (DIS, red triangles) versus healthy donors (HD, black triangles). Top contributing variables to the two principal components are displayed, highlighting subsets with the highest discriminatory power. **(B)** Frequencies and marker expression levels of selected immune cell populations significantly altered in CeAD including increased CD28 expression on naïve CD8 ⁺ T cells, elevated CD8 ⁺ DNAM-1 ⁺ T cells, NKp46 ⁺ NK cells, CD25 ⁺ mDC2 cells, and GrK ⁺ CD8 ⁺ T cells. Tregs and NKT cells showed non-significant reductions. **(C)** Effect size and fold-change of selected parameters, with functionally validated markers highlighted in bold. **(D)** Composite immune-activation Score (CS) derived from top-ranked features: CD8 naïve CD28 ⁺ MFI × NKp46 ⁺ NK MFI × CD141 ⁺ mDC2 CD25⁺ × CD8 ⁺ GrK⁺ × NKT cell frequency. The CS robustly discriminated CeAD from controls (left, ***p < 0.001), achieving perfect classification in ROC analysis (right, AUC = 1.00, 100% sensitivity and specificity at threshold = 1.8). Data represent mean ± SD; *p < 0.05, **p < 0.01, ***p < 0.001 (Kruskal-Wallis + Dunn’s post-test).

To identify immune cell parameters with the greatest discriminatory value, sparse Partial Least Squares Discriminant Analysis (sPLS-DA) was performed, focusing on the 20 most relevant immune parameters. This approach demonstrated a clear separation of CeAD patients and healthy donors, independent of stroke status (Fig 2A). Key drivers of separation included increased expression of CD28 on naïve CD8 ⁺ T cells, NKp46 on NK cells, and IL-2Rα (CD25) on mDC2 cells (Fig 2A). These alterations may indicate increased activation potential within CD8⁺ and NK cell compartments; however, this interpretation remains tentative given the limited functional validation. Exploratory univariate plots (Fig 2B) confirmed significantly elevated expression of CD8 ⁺ CD28 ⁺ , NKp46 ⁺ NK cells, and CD25 ⁺ mDC2 cells in CeAD. Additionally, a trend toward reduced frequencies of regulatory T cells (Tregs) and NKT cells was observed, although these differences did not reach statistical significance. Increased granzyme K expression in naïve CD8 ⁺ T cells indicated a shift toward cytotoxic potential, while memory populations showed no significant changes. Effect size analyses (Fig 2C) highlighted IL-2Rα (CD25) on mDC2 cells, naïve CD8 ⁺ T cells, and granzyme K-expressing naïve CD8 ⁺ T cells as parameters with both statistical significance and robust magnitude of difference (fold change >1.2 or <0.8). Based on these high-impact features, we derived a composite immune activation score (Fig 2D), which provided clear segregation of CeAD and controls in this cohort. Receiver operating characteristic (ROC) analysis identified an optimal cut-off score (1.8), yielding perfect classification within this small sample.

### Metagenomic screening in CeAD patients

To explore a potential infectious contribution, untargeted metagenomic sequencing was performed in peripheral blood samples from four CeAD patients, yielding 24–36 million reads per sample. No known viral, bacterial, or fungal pathogens were detected. Given that sampling occurred shortly after the dissection event, at a time when any preceding infection would likely have already resolved, the absence of detectable pathogens does not exclude a role for infection in CeAD pathogenesis.

## Discussion

This case series provides the first comprehensive immune profiling of peripheral blood in patients with CeAD. We identified a specific immune activation signature marked by increased cytotoxic potential and diminished regulatory features. These findings suggest that CeAD is associated with distinct peripheral immune alterations, raising important questions regarding their origin and pathogenic relevance.

Two principal, non-mutually exclusive mechanisms may explain our observations. On the one hand, immune activation could precede dissection as a pathogenic driver. Prior studies have linked recent, particularly viral, infections to CeAD onset [7–9], supporting the concept of a post-infectious or autoimmune-mediated trigger. In line with this, we observed increased CD28 expression on naïve CD8 ⁺ T-cells, elevated NKp46 expression on NK cells, and enhanced IL-2Rα on mDC2 cells, together pointing toward a proinflammatory and cytotoxic-prone immune state. The concomitant upregulation of granzyme K in naïve CD8 ⁺ T-cells further supports a cytokine-driven effector readiness. Similar immune alterations have been described in systemic autoimmune diseases, including systemic lupus erythematosus and rheumatoid arthritis [17], where CD28 ⁺ granzyme K-high, perforin-low CD8 ⁺ T-cells infiltrate inflamed tissues and secrete proinflammatory cytokines such as IFN-γ [17]. Likewise, NKp46 overexpression has been associated with augmented cytotoxicity and disease severity in autoimmune contexts [18]. Collectively, these parallels raise the possibility that CeAD shares immune signatures with systemic autoimmune and inflammatory disorders.

On the other hand, the immune alterations in CeAD may represent a secondary consequence of endothelial disruption. Arterial dissection exposes subendothelial structures that can elicit a coordinated immune response, as shown in aortic dissection where macrophage infiltration, T-cell activation, NK-cell cytotoxicity, and B-cell responses collectively create a proinflammatory milieu within the vessel wall [19]. Such localized vascular inflammation may extend into the peripheral circulation, giving rise to the systemic immune profile observed here.

In this cohort including CeAD patients with and without ischemic stroke, the identified immune activation pattern remained largely consistent. The overall immune profile was dominated by enhanced cytotoxic potential and reduced regulatory features, independent of stroke occurrence. This suggests that the observed immune alterations are primarily related to the dissection event rather than the ischemic complication. Available evidence indicates that CeAD accompanied by ischemic stroke is associated with higher plasma levels of hepatocyte growth factor (HGF) and stromal cell-derived factor 1α (SDF-1α) as well as lower IL-4 concentrations, reflecting a more pronounced systemic inflammatory activation [20]. In addition, increased titers of anti-collagen type I antibodies have been reported in CeAD and, even more markedly, in stroke of other etiologies [21]. These observations support the notion that ischemic stroke triggers an additional inflammatory response, characterized by enhanced cytokine release and extracellular matrix remodeling. In our study, however, the core CeAD-related immune signature persisted across patients with and without stroke, suggesting that the dissection itself, rather than subsequent ischemia, represents the main determinant of the observed immune phenotype.

The strengths of our study include carefully age- and sex-matched controls, standardized immunophenotyping, and sampling during the subacute phase, thereby minimizing technical and temporal variability. Comprehensive clinical characterization and systematic exclusion of infections, vaccinations, medication use, and major comorbidities across all study groups further reduce potential confounding and strengthen the validity of our findings. Nevertheless, several limitations must be acknowledged. The small sample size and single-center design restrict statistical power and generalizability; thus, the study should be considered exploratory. The cross-sectional design precludes conclusions on causality; therefore, larger multicenter cohorts with longitudinal sampling, including patients with and without stroke as well as disease controls with other vascular pathologies, will be essential to determine whether immune alterations precede or follow arterial dissection and to validate the specificity of the observed immune signature. Although blood sampling was performed within a defined subacute window (6–14 days post-onset), minor temporal variability cannot be fully excluded, even though correlation analyses did not reveal systematic effects. Despite systematic screening for infections, vaccinations, medication use, and comorbidities across all study groups, residual confounding due to subclinical or lifestyle-related factors cannot be entirely ruled out. While pre-event sampling would be ideal to identify potential infectious triggers, such an approach is infeasible in spontaneous CeAD due to the unpredictable disease onset. Future studies including CeAD patients with and without stroke, as well as disease controls with other vascular pathologies, will be essential to validate the specificity and reproducibility of the identified immune activation signature. If confirmed, the observed immune alterations may serve as biomarkers or therapeutic targets, guiding the exploration of immunomodulatory strategies in preclinical models or early-phase clinical trials. The ROC analysis was conducted in a small exploratory cohort, and results should therefore be interpreted with caution and validated independently in larger, multicenter studies.

## Conclusions

In conclusion, our findings demonstrate that CeAD is associated with a distinct peripheral immune signature. Whether this signature reflects a post-infectious autoimmune trigger or secondary immune-inflammatory response to endothelial injury remains yet unresolved. The preservation of this immune signature in patients with and without ischemic stroke indicates that it primarily reflects the dissection-related immune response rather than stroke-specific changes. Future multicenter studies with larger cohorts and longitudinal designs are needed to clarify whether immune alterations contribute to CeAD pathogenesis, predict recurrence and vascular healing, and serve as potential therapeutic targets.

## Supporting information

S1 Fig Sex-stratified analysis of major immune cell subsets. Percentages of lymphocytes, monocytes, CD4 ⁺ T cells, CD8 ⁺ T cells, B cells, NK cells, NKT cells, and myeloid dendritic cells (mDCs) were analyzed separately for female and male participants in healthy donors (HD, black/gray triangles) and CeAD patients (red triangles). While minor variations were observed between sexes, the principal differences, higher CD4 ⁺ T cell frequencies and reduced NKT cells in CeAD, remained consistent in both female and male subgroups. Bars represent mean ± SD.
(TIF)

S2 Fig Sex-stratified analysis of immune activation markers and functional parameters. Expression levels of CD28 on naïve CD8 ⁺ T cells, NKp46 on NK cells, CD25 on mDC2, and additional activation or cytotoxicity-associated markers (e.g., granzyme K, DNAM-1, CD107a) were assessed in female and male subgroups of CeAD and HD participants. The CeAD-associated immune activation pattern was preserved in both sexes, indicating that the observed immune signature is independent of sex distribution. Bars represent mean ± SD.
(TIF)
