# Supplementary figures and images for: Spontaneous cervical artery dissection is associated with a distinct peripheral immune cell signature
Source: PLoS One. 2026 Jan 22;21(1):e0340592. doi: 10.1371/journal.pone.0340592 (PMC12826460; doi:10.1371/journal.pone.0340592)

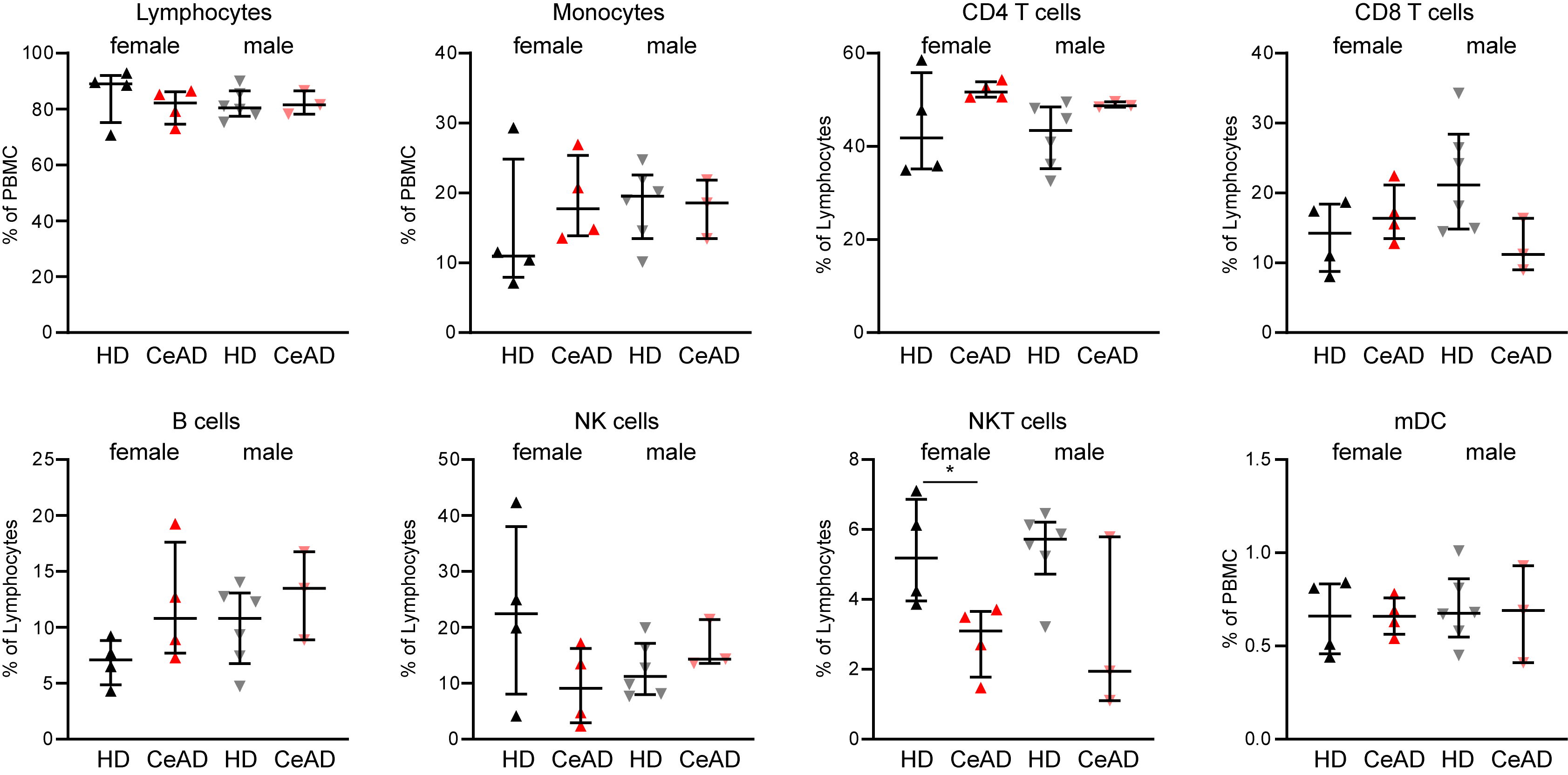

Supplement: S1 Fig — Percentages of lymphocytes, monocytes, CD4 ⁺ T cells, CD8 ⁺ T cells, B cells, NK cells, NKT cells, and myeloid dendritic cells (mDCs) were analyzed separately for female and male participants in healthy donors (HD, black/gray triangles) and CeAD patients (red triangles). While minor variations were observed between sexes, the principal differences, higher CD4 ⁺ T cell frequencies and reduced NKT cells in CeAD, remained consistent in both female and male subgroups. Bars represent mean ± SD. (TIF) [file pone.0340592.s001.tif]

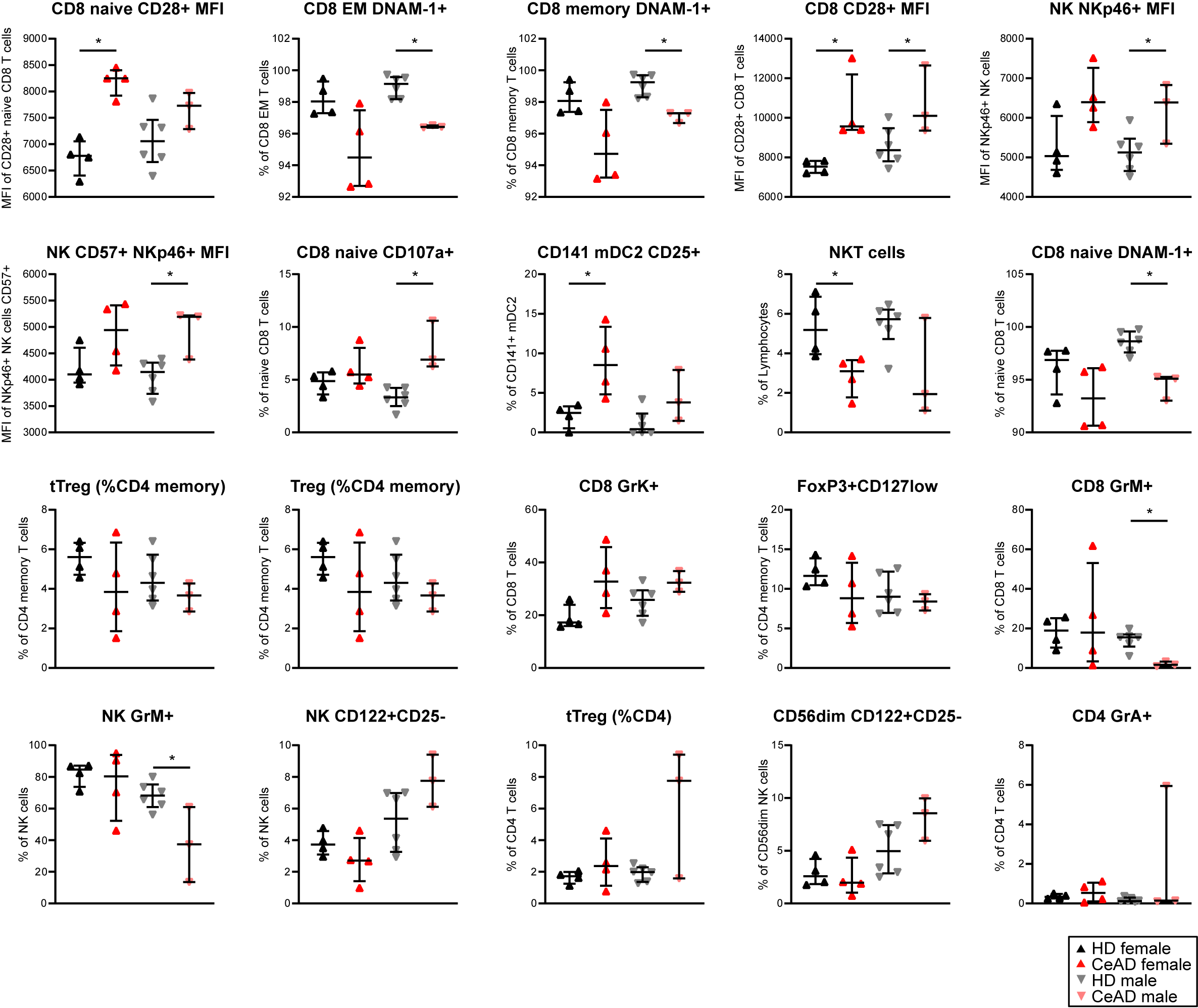

Supplement: S2 Fig — Expression levels of CD28 on naïve CD8 ⁺ T cells, NKp46 on NK cells, CD25 on mDC2, and additional activation or cytotoxicity-associated markers (e.g., granzyme K, DNAM-1, CD107a) were assessed in female and male subgroups of CeAD and HD participants. The CeAD-associated immune activation pattern was preserved in both sexes, indicating that the observed immune signature is independent of sex distribution. Bars represent mean ± SD. (TIF) [file pone.0340592.s002.tif]
